# Association between Care-need Level after Discharge and Long-term Outcomes in 7491 Patients Requiring Rehabilitation for Stroke

**DOI:** 10.31662/jmaj.2023-0094

**Published:** 2023-11-16

**Authors:** Takaaki Konishi, Haruhi Inokuchi, Yusuke Sasabuchi, Hiroki Matsui, Masahiko Tanabe, Yasuyuki Seto, Hideo Yasunaga

**Affiliations:** 1 Department of Breast and Endocrine Surgery, The University of Tokyo, Tokyo, Japan; 2 Department of Clinical Epidemiology and Health Economics, The University of Tokyo, Tokyo, Japan; 3 Department of Rehabilitation Medicine, The University of Tokyo Hospital, Tokyo, Japan; 4 Data Science Center, Jichi Medical University, Tochigi, Japan

**Keywords:** bedridden, long-term care insurance, rehabilitation, stroke

## Abstract

**Introduction:**

Stroke is a major cause of disability and mortality worldwide and requires long-term care, including rehabilitation. This study aims to elucidate the association between care-need levels after discharge and long-term outcomes in patients with stroke.

**Methods:**

We used a Japanese administrative database that covers both medical and long-term care insurance systems to retrospectively identify 7491 patients who underwent acute-phase in-hospital rehabilitation for stroke between June 2014 and February 2019. We investigated the association between nationally standardized care-need levels (support levels 1-2 and care-need levels 1-3) 6 months after discharge and long-term outcomes. Using the Fine-Gray model, we conducted multivariable survival analysis with adjustment for patient backgrounds and treatment courses to estimate hazard ratios (HR) for mortality and the incidence of being bedridden.

**Results:**

The median age was 82 (interquartile range [IQR], 76-87) years, 5418 patients (72%) had cerebral infarction, and 4009 patients (54%) had partial dependence after discharge. During a median follow-up of 580 (IQR, 189-925) days, 1668 patients (22%) became bedridden, and 2174 patients (29%) died. Compared with patients with support level 1, those with higher care-need levels showed significantly higher proportions of being bedridden―the subdistribution HR [95% confidence interval] were 1.52 [1.10-2.12], 2.85 [2.09-3.88], and 3.79 [2.79-5.15] in those with care-need levels 1, 2, and 3, respectively. Higher care-need levels were also significantly associated with higher mortality.

**Conclusions:**

This large-scale observational study demonstrated that a higher level of care-need after discharge was significantly associated with poorer functional outcomes and higher mortality.

## Introduction

Stroke is a major cause of mortality and disability worldwide, with significant socioeconomic burden, which is projected to increase in the future because of the demographic transitions of populations ^(1)^. Stroke survivors often require short- and long-term rehabilitation to improve their activities of daily living and reduce the occurrence of pressure sores and deep vein thrombosis ^(2), (3), (4), (5), (6)^. To provide long-term nursing services such as rehabilitation, a universal long-term care insurance system was established in 2000 in Japan ^(7), (8), (9), (10)^. Patients can receive long-term services based on a certificate that states the need for long-term care in accordance with their activities of daily living ^(10), (11)^. Indeed, in Japan, after being discharged from acute and rehabilitation hospitals where the medical insurance system is applicable, patients with stroke can generally receive long-term rehabilitation (home-based and ambulatory) using the long-term care insurance system ^(12), (13), (14), (15)^.

Although functional outcomes and survival are important for patients with stroke, limited evidence regarding real-world long-term outcomes is available. Previous cohort studies have reported risk factors for poor long-term survival in stroke survivors ^(16), (17), (18), (19), (20)^. A Taiwanese retrospective study involving 7767 patients reported that rehabilitation within the first 3 months after stroke admission was significantly associated with low mortality for 10 years ^(20)^. A European multi-institutional study of 532 patients reported that old age, some comorbidities, and a low Barthel index score at 6 months after stroke were significantly associated with poor 5-year mortality ^(17)^. However, these studies did not report functional outcomes despite their clinical importance. In addition, because there are few databases covering both medical and long-term care, large-scale studies have seldom been conducted. A large-scale study using real-world data can provide representative evidence for very large patient populations ^(21)^.

Hence, we aimed to investigate the association between the degree of activity of daily living after discharge and the long-term outcomes in patients with stroke using a large-scale Japanese administrative database that covers both universal medical and long-term care insurance systems ^(22), (23)^.

## Materials and Methods

### Data source

We conducted this retrospective cohort study using an administrative database obtained from municipalities in Tochigi Prefecture, a northern prefecture of the Greater Tokyo Area ^(23), (24), (25)^. Out of 25 municipalities (14 cities and 11 towns), 18 agreed to submit anonymous claims data for research. The database contained claims for two medical insurances and a long-term care insurance for approximately 700,000 residents. As medical insurances, the data on National Health Insurance (for self-employed individuals, retired individuals, and their dependents) and Late Elders’ Health Insurance (for all people aged 75 years or older) are included. Since a majority of people in Japan has been publicly insured for medical care since 1961 ^(26), (27)^, almost all residents aged 65 years or older in the 18 districts were included in the database. In addition, the Japanese government implemented a mandatory public long-term care insurance in 2000 ^(7), (8), (9), (10)^. Residents aged 65 years or older are regarded as primary insured candidates, and residents aged 40-64 years who were diagnosed with one of the 16 predetermined diseases, including stroke, are regarded as secondary insured candidates. Of these candidates, residents who meet the eligibility criteria can receive long-term care insurance. Accordingly, this database contains the following patient-level data for hospitalization and outpatient visits: year and month of birth, sex, diagnoses recorded with the International Classification of Diseases, 10th revision (ICD-10) codes, interventions and surgical procedures, care-need level, long-term care services, and death.

A care-need level is required to receive long-term care insurance services, which is assessed by a nationally standardized certification system, irrespective of the income level and availability of informal care provided by the family ^(7), (10)^. First, a trained local government official visits a candidate (at home or at the hospital) to evaluate their nursing care needs using a questionnaire of approximately 90 items on the current physical and mental status and to survey recent use of medical services (e.g., tubal feeding, hemodialysis, and decubitus care) with a detailed note. Concurrently, the primary care physician fills out a paper-based statement on the candidate’s condition in a common format. Next, the results of the questionnaire and part of the statement are computed to assign the candidate to one of the seven care-need levels: support levels 1-2 and care-need levels 1-5. Finally, the Nursing Care Needs Certification Board―which includes physicians, nurses, and other experts in health and social services appointed by a mayor―decides the final care-need level that determines the service benefits covered by the long-term care insurance after taking into consideration the officer’s note and the primary care physician’s statement. In principle, the care-need level is re-evaluated once or twice a year ^(10)^. A previous study reported that the level correlates highly with the Barthel index ^(11)^; support levels 1-2 and care-need level 1 were comparable with the Barthel index scores of 85-95 (independent with minor assistance), care-need levels 2-3 were comparable with the Barthel index scores of 65-80 (partial dependence), and care-need levels 4-5 were comparable with the Barthel index scores of <40 (complete dependence, bedridden).

### Study protocol

We retrospectively identified patients aged 40 years or older who underwent rehabilitation during acute-phase hospitalization for stroke (cerebral infarction [*ICD-10* code: I63], intracerebral hemorrhage [I61], and subarachnoid hemorrhage [I60]) between June 2014 and February 2019, using the abovementioned administrative database. We excluded patients who (i) were included in the database within 6 months preceding admission (a window period to obtain information on comorbidities), (ii) had care-need levels 4-5 after 6 months of discharge (because they would not be able to experience the bedridden status afterward), (iii) died within 6 months after discharge (because they would not experience the outcomes afterward), and (iv) had no certification of the care-need level within 6 months after discharge (to exclude patients with very mild stroke, those who are unwilling to avail public care services, and those who moved to another prefecture). We categorized eligible patients into five groups according to their care-need level soon after discharge: support levels 1-2 and care-need levels 1-3. We defined the care-need level soon after discharge as 6 months after discharge since the care-need level would be determined within 6 months after discharge and would not change for the first 6 months ^(7)^.

The primary and secondary outcomes were, respectively, the incidence of a bedridden status (care-need levels 4-5) and mortality >6 months after discharge. We examined patients’ background factors, including patient characteristics (sex, age, and comorbidities), type of stroke (cerebral infarction, intracerebral hemorrhage, and subarachnoid hemorrhage), and treatment course (hospital day of initiating rehabilitation, total length of hospital stay, and rehabilitation after discharge). Regarding age, we deemed patients to have been born on the first day of the month and calculated their age at admission. We categorized the age into six groups: 40-69, 70-74, 75-79, 80-84, 85-89, and 90 years or older. Comorbidities were assessed using the Charlson comorbidity index, which was defined on the basis of ICD-10 codes within the window period (6 months preceding admission); for example, the index included previous cerebrovascular diseases and preexisting hemiplegia/paraplegia as its factors ^(28), (29)^. Hospital days of initiating rehabilitation were categorized into three groups: ≤2 days, 3-7 days, and >7 days. The total length of hospital stay was categorized into four groups: ≤14 days, 15-30 days, 31-90 days, and >90 days. We defined rehabilitation after discharge as home-based or ambulatory rehabilitation within 6 months after discharge under medical or long-term care insurance.

The requirement for informed consent in the present study was waived because of the anonymity of the patient database. This study is in accordance with the Strengthening the Reporting of Observational Studies in Epidemiology statement ^(30)^ and the Act on the Protection of Personal Information. The Institutional Review Board of Jichi Medical University comprehensively approved clinical epidemiological studies using the current administrative claims database (approval number 22-202; April 14, 2023).

### Statistical analysis

First, we plotted the Kaplan-Meier curve for outcomes stratified by the care-need level after discharge. Follow-up started after discharge and ended at the incidence of outcomes, exit from the database, or in February 2019, whichever occurred first.

Second, we conducted a multivariable survival analysis to investigate the association between the care-need level after discharge and the outcomes with adjustment for the aforementioned background factors (patient characteristics, type of stroke, and treatment course). Hazard ratios (HR) were obtained as estimates of the relative risk of outcomes using Cox proportional hazard regression models. In the analysis for the incidence of a bedridden status, we used a Fine-Gray subdistribution hazard model, where mortality was regarded as a competing risk ^(23), (31), (32)^.

All 95% confidence intervals (CI) and *p*-values were based on two-sided hypothesis tests, where *p* < 0.05 is considered statistically significant. We conducted statistical analyses using Stata/SE 17.0 (StataCorp, College Station, TX, USA).

## Results

We identified 20,077 patients aged 40 years or older who underwent rehabilitation during acute-phase hospitalization for stroke between June 2014 and February 2019. We excluded 12,586 patients who (i) had insurance within the window period (n = 5066), (ii) had care-need levels 4-5 after 6 months of discharge (n = 1539), (iii) died within 6 months after discharge (n = 212), and (iv) had no certification of care-need level within 6 months after discharge (n = 5769).

Table 1 shows the background factors and care-need level after discharge of the eligible 7491 patients. The mean age was 82 (interquartile range [IQR], 76-87) years. Regarding the type of stroke, cerebral infarction was most common (72%), followed by intracerebral hemorrhage (24%) and subarachnoid hemorrhage (3.8%). The mean hospital day of starting rehabilitation was 3 (IQR, 2-6) days, and the mean total length of hospital stay was 30 (IQR, 16-72) days. Approximately 40% of the patients started rehabilitation within 2 days of admission, and 60% continued rehabilitation after discharge. Six months after discharge,12% of patients were assigned to support level 1, 11% to support level 2, 23% to care-need level 1, 26% to care-need level 2, and 28% to care-need level 3.

**Table 1.** Background Factors and Care-Need Level after the Discharge of 7491 Patients Who Underwent Rehabilitation during Hospitalization for Stroke.

Variables	n	(%)	Variables	n	(%)
**Patient characteristics**			**Type of stroke**		
Male sex	3644	(49)	Cerebral infarction	5418	(72)
Age category			Intracerebral hemorrhage	1791	(24)
40-69	724	(9.7)	Subarachnoid hemorrhage	282	(3.8)
70-74	786	(10)			
75-79	1435	(19)	**Treatment course**		
80-84	1873	(25)	Hospital days of initiating rehabilitation		
85-89	1681	(22)	≤2 days	2776	(37)
≥90	992	(13)	3-7 days	3216	(43)
Charlson comorbidity index			>7days	1499	(20)
1	1129	(15)	Total length of hospital stay		
2	1306	(17)	≤14 days	1461	(20)
3	1281	(17)	15-30 days	2342	(31)
4	1132	(15)	31-90 days	2253	(30)
5	830	(11)	>90 days	1435	(19)
6	565	(7.5)	Rehabilitation after discharge^*^		
7	426	(5.7)	Medical insurances use	2935	(39)
8	239	(3.2)	Long-term care insurance use	1447	(19)
9	169	(2.3)			
10	127	(1.7)	**Care-need level after discharge**†		
11	100	(1.3)	Support level 1	910	(12)
12	61	(0.8)	Support level 2	812	(11)
13	50	(0.7)	Care-need level 1	1760	(23)
14	29	(0.4)	Care-need level 2	1937	(26)
≥15	47	(0.6)	Care-need level 3	2072	(28)

^*^Home-based or ambulatory rehabilitation conducted within 6 months of discharge. †Care-need level 6 months after discharge

**Figure 1.**
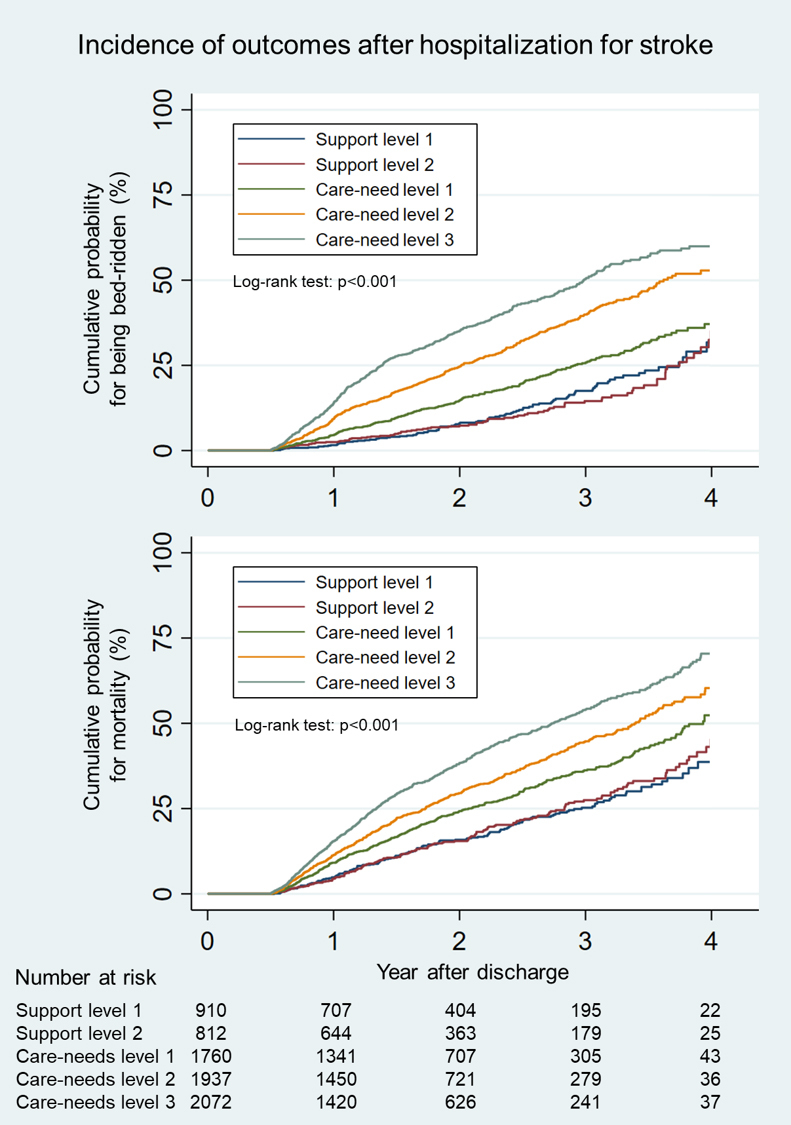
Kaplan-Meier curves for outcomes after hospitalization for stroke categorized by the five care-need levels after discharge. The care-need level after discharge was defined at 6 months after discharge. The curves after 4 years are not depicted because of the small number of cases.

Figure 1 shows the Kaplan-Meier curve for outcomes categorized by the five care-need levels after discharge. The median follow-up period was 580 (IQR, 189-925) days. Patients with support levels 1-2 demonstrated similar curves. Compared with these patients, those with care-need levels 1-3 showed a consistently higher incidence of outcomes.

Table 2 shows the HR of the care-need level after discharge for being bedridden. Overall, 1668 patients (22%) became bedridden. As the care-need level increased, patients became bedridden more frequently: support level 1 (10%), support level 2 (11%), care-need level 1 (17%), care-need level 2 (26%), and care-need level 3 (33%). Multivariable survival analysis showed that the patients with care-need levels 1-3 had significantly higher incidences of being bedridden than those with support level 1: the subdistribution HR (95% CI) for care-need levels 1, 2, and 3 were 1.52 (1.10-2.12), 2.85 (2.09-3.88), and 3.79 (2.79-5.15), respectively.

**Table 2.** Hazard Ratios of the Care-Need Level after Discharge for the Incidence of Being Bedridden in Patients Who Underwent Rehabilitation during Hospitalization for Stroke.

Care-need level after discharge*	Outcome†		SHR†	95% CI	P-value
	n	(%)			
Support level 1	95	(10)	Reference		
Support level 2	88	(11)	1.04	0.70-1.55	0.83
Care-need level 1	292	(17)	1.52	1.10-2.12	0.012
Care-need level 2	505	(26)	2.85	2.09-3.88	<0.001
Care-need level 3	688	(33)	3.79	2.79-5.15	<0.001

Abbreviations: CI, confidence interval; SHR, subdistribution hazard ratio *Care-need level 6 months after discharge †Hazard ratios for the outcome (being bedridden [care-need level 4-5] >6 months after discharge) were calculated using multivariable survival analysis with adjustment for background factors. We used the Fine-Gray subdistribution hazard model, in which mortality was regarded as a competing risk.

The overall mortality during the study period was 29%. Compared with patients with support levels 1-2, those with care-need levels 1-3 showed higher mortality (care-need level 1, 26%; care-need level 2, 31%; and care-need level 3, 37%) (Table 3). Multivariable survival analysis also demonstrated that patients with care-need levels 1-3 had significantly higher incidences of mortality than those with support level 1: HR (95% CI) of care-need levels 1, 2, and 3 were 1.47 (1.23-1.77), 1.86 (1.56-2.21), and 2.56 (2.15-3.04), respectively.

**Table 3.** Hazard Ratios of the Care-Need Level after Discharge for Mortality in Patients Who Underwent Rehabilitation during Hospitalization for Stroke.

Care-need level after discharge*	Outcome†		HR†	95% CI	P-value
	n	(%)			
Support level 1	164	(18)	Reference		
Support level 2	161	(20)	0.99	0.80-1.23	0.96
Care-need level 1	466	(26)	1.47	1.23-1.77	<0.001
Care-need level 2	608	(31)	1.86	1.56-2.21	<0.001
Care-need level 3	775	(37)	2.56	2.15-3.04	<0.001

Abbreviations: CI, confidence interval; HR, hazard ratio *Care-need level 6 months after discharge †Hazard ratios for the outcome (mortality >6 months after discharge) were calculated using multivariable survival analysis with adjustment for background factors.

## Discussion

This current retrospective cohort study used an administrative claims database, and we investigated the association between the care-need level after discharge and long-term prognosis in 7491 patients with stroke. Kaplan-Meier curves showed that patients with a higher care-need level had a higher incidence of being bedridden and higher mortality. Multivariable survival analysis with adjustment for background also demonstrated a significantly higher HR for a higher care-need level for a poorer long-term prognosis.

The current study mainly included patients with cerebral infarction, and the remaining patients had hemorrhage. This distribution was similar to that in previous retrospective studies on rehabilitation for stroke ^(18), (19), (33), (34)^. Nevertheless, the mean age of the patients was higher in the current study compared with those of the previous studies, presumably because of the nature of the current database; that is, the database included almost all residents aged 65 years or older but did not include residents aged 40-64 years who joined the employee’s insurance (insurance other than the National Health Insurance). Thus, the Charlson comorbidity index and the incidence of being bedridden and mortality might have been higher than those of general Japanese patients with stroke.

Although the long-term functional outcome of stroke has rarely been described, the current analysis revealed a significant association between activities of daily living soon after discharge and being bedridden during the observation period. We consider that the result was statistically correct because we used a Fine-Gray model to adjust for competing risk ^(31), (32)^; that is, because a conventional method for survival analysis is based on the assumption of noninformative censoring, informative censoring caused by competing events (for instance, mortality in the current analysis) can skew the results of conventional survival analysis for the primary outcome (being bedridden in the current analysis) ^(23)^. In addition, in the current multivariable survival analysis, we adjusted for important background factors (age, comorbidities, type of stroke, and treatment course) that were specified in previous studies on long-term survival ^(16), (17), (18), (19), (20)^. The results indicate that patients at discharge who depend on nursing care, even partially, are at high risk of being bedridden despite the existence of long-term care after discharge. To achieve a good long-term functional outcome, patients and clinicians may have to aim for support levels 1 and 2 (independence with minor assistance) of rehabilitation during hospitalization. Moreover, extensive rehabilitation after discharge can help avoid being bedridden ^(5), (6)^. Further, long-term care insurance services may improve the accessibility of patients to rehabilitation.

Impaired activities of daily living soon after discharge are associated with long-term mortality. This corresponds with a previous multi-institutional study, which used a multivariable analysis to show that an impaired Barthel index 6 months after stroke was associated with poor long-term prognosis ^(17)^. However, the study did not include poststroke rehabilitation after discharge as an explanatory variable, although rehabilitation could improve the long-term prognosis ^(20)^. Since a significant association between activities of daily living soon after discharge and long-term mortality was observed even with adjustment for rehabilitation after discharge, we consider that the association would be robust. Therefore, functional gain during hospitalization may be essential for long-term survival, as reported in a previous study ^(18)^. Furthermore, activities of daily living (such as care-need level) after discharge can be used as an explanatory variable to adjust for stroke severity in a longitudinal retrospective study.

Although a previous study reported the correlation between the care-need level and the Barthel index ^(11)^, the association between the level and long-term clinical outcomes has rarely been investigated. To our knowledge, no studies demonstrated long-term outcomes following stroke based on the care-need level, although a single-institutional study investigated in-hospital outcomes ^(35)^. While previous studies evaluated the preexisting care-need level before hospitalization ^(24), (25), (35)^, we evaluated the care-need level after hospitalization. Furthermore, the current study demonstrates a much longer prognosis than the other studies. Even though the care-need level was assessed and determined for the allocation of care resources in the subacute or recovery phase (soon after the discharge), the cared-need level potentially allows for the prediction of functional and survival prognosis in the chronic phase following stroke. Since the care-need level is determined according to a nationally standardized certification system ^(7), (8), (9), (10)^, the current results can benefit clinical decisions, epidemiological studies, and health policies for patients with stroke all across Japan.

This study had several limitations. First, because the long-term care insurance covers residents aged 40 years or older, patients aged <40 years with stroke were not included in the analysis. However, since the majority of patients with stroke worldwide are elderly ^(35), (36)^, we consider that the results have generalizability. Second, the diagnosis of stroke in the claims data may have a low accuracy. To improve specificity, we included patients who underwent rehabilitation for stroke in the analysis, as recommended in a previous Japanese validation study on stroke ^(37)^. Third, we did not obtain information on the severity of stroke (for instance, the National Institutes of Health Stroke Scale) at admission. Indeed, activities of daily living after discharge would reflect the severity of stroke at admission ^(38)^. Based on the current study, however, we consider that the degree of activities of daily living after discharge reliably predicts long-term outcomes regardless of severity at admission. Additionally, we were unable to obtain information on simple rehabilitation as a part of nursing and family care and adjust for it in the analysis. However, these factors may have limited effects on outcomes compared with the qualified rehabilitation conducted by nationally certified therapists. Fourth, we did not take the frequency of rehabilitation into account. Because the frequency can vary widely during the long observation period, we were unable to determine an objectively reasonable definition of the frequency. Instead, we used the initial day of rehabilitation, the length of hospital stay (presumably mainly for rehabilitation), and the insurance type for rehabilitation as objective background factors. Furthermore, because the frequency of rehabilitation after the allocation of the care-need level can be determined based on the level, the frequency after discharge was an intermediate factor not to be adjusted in the analysis ^(39)^. Finally, because the care-need level in the long-term care insurance services is specific to Japan and not a universal concept, the generalizability to other countries may be limited ^(24), (25)^. Additionally, even though the decision process for the care-need level was nationally standardized, the determined level might be inconsistent, and available care services can vary among municipalities in Japan ^(10)^. However, the care-need level reflected the Barthel index very well ^(11)^, enhancing the generalizability of the level ^(24)^.

In conclusion, this large retrospective observational study that used an administrative database in Japan showed an association between the care-need level 6 months after discharge and the long-term outcomes in patients who underwent rehabilitation during hospitalization for stroke. Impaired activities of daily living soon after discharge were significantly associated with poorer functional outcomes and higher mortality. To achieve good long-term outcomes, patients and clinicians should aim for independence with minimal assistance in rehabilitation during hospitalization.

## Article Information

### Conflicts of Interest

Takaaki Konishi received grants from Pfizer Co. Ltd., Kanzawa Medical Research Foundation, and Japan Kampo Medicines Manufacturers Association outside the submitted work.

### Sources of Funding

This work was supported by grants from the Ministry of Health, Labour and Welfare, Japan grant number 21AA2007 and the Ministry of Education, Culture, Sports, Science and Technology, Japan grant number 19K19394.

### Author Contributions

TK and YS were responsible for the concept and design of the study; YS, HM, and HY collected and assembled the data; TK and YS analyzed the data; IH, YS, MT, YS, and HY contributed to the interpretation of the analyses; TK wrote the initial draft; and IH, YS, HM, MT, YS, and HY provided critical revision of the manuscript for important intellectual content.

### Approval by Institutional Review Board (IRB)

This study was approved by the Institutional Review Board of The Jichi Medical University (approval number 22-202; April 14, 2023).

### Disclaimer

Yasuyuki Seto is one of the Editors of JMA Journal and on the journal’s Editorial Staff. He was not involved in the editorial evaluation or decision to accept this article for publication at all.
